# NEIGHBOUR-IN: Image processing software for spatial analysis of animal grouping

**DOI:** 10.3897/zookeys.515.9390

**Published:** 2015-07-30

**Authors:** Yves Caubet, Freddie-Jeanne Richard

**Affiliations:** 1 Université de Poitiers - Faculté des Sciences, UMR CNRS 7267 EBI – “Écologie, Évolution, Symbiose”, Bat. B8-B35; 6, rue Michel Brunet, TSA 51106, F-86073 POITIERS Cedex 9, France

**Keywords:** Inter-individual distances, aggregation, individual attraction, spatial distribution

## Abstract

Animal grouping is a very complex process that occurs in many species, involving many individuals under the influence of different mechanisms. To investigate this process, we have created an image processing software, called NEIGHBOUR-IN, designed to analyse individuals’ coordinates belonging to up to three different groups. The software also includes statistical analysis and indexes to discriminate aggregates based on spatial localisation of individuals and their neighbours. After the description of the software, the indexes computed by the software are illustrated using both artificial patterns and case studies using the spatial distribution of woodlice. The added strengths of this software and methods are also discussed.

## Introduction

Group formation or crowd formation behaviour occurs in many taxa, from very simple organisms (bacteria) to highly complicated organisms (e.g. mammals), both wild and domestic (Krause and Ruxton 2002; Parrish and Edelstein-Keshet 1999; Parrish and Hamner 1997). Depending on the species, crowds of individuals are referred to as a herd in mammals, flocks in birds, schools in fish, swarms in insects and many other terms that indicate aggregation. Species can be range to simply aggregated species with temporal fluid group composition to very complex and relatively stable composition with non-random structures (Kutsukake 2009).

Group living confers several advantage compare to solitary lifestyle and many animals live in groups for part or all of their lives. Aggregate can be the results of abiotic factors and environmental heterogeneity (non-social aggregates) or relied on mutual attraction (interattraction) resulting in different formation process (Broly et al. 2013). In most of the cases, the ecological and social factors that explain group life are unknown. Ecological factors favouring group life are numerous and usually addressed the benefits from individuals association or differences in fitness related with spatial position of individuals in groups (Krause and Ruxton 2002). The main two factors are the availability of food and the presence of predators and the optimum group size change according to the species and environmental pressure variations. For example, group’s dimension and geographical repartition change according of both predators and food availability in starlings (Zoratto et al. 2009). Group size reduces the risk of predation (Brown and Brown 1987) and offers a better foraging efficiency (Stacey 1986) and large groups present feeding advantage compared to small groups (Miller and Dietz 2006). Little is known in general about how group size affects individual welfare (Ohl and Putman 2014). Costs are mainly the result of competition for limited resources (food, mate, habitat...) and increase in group size favours disease transmission, probability of infection and ectoparasitism that affect survival (Brown and Brown 2004; Krause and Ruxton 2002).

Animal group formation is a complex dynamic system made up of potentially thousands of individuals. The group formation is the result of each individual’s behaviour under the influence of many (and non-exclusive) parameters, such as heterogeneities of environment, inter-individual interactions, and temporal changes (ie, season, reproduction, and feeding) (Camazine et al. 2003; Sumpter and Pratt 2009). In cockroaches, the aggregation relies on mechanisms of amplification depending on the interactions with other individuals (Jeanson et al. 2005). Group dynamic is also the result of the heterogeneous social relationships and conflict management for maintaining group living in mammals (Kutsukake 2009). Moreover, animal groups exhibit different patterns according to the species (Parrish and Edelstein-Keshet 2000; Parrish and Edelstein-Keshet 1999) which necessitate incorporating species characteristics for conceptual questions and modelling.

To better understand group dynamic complexity, the identification of factor influencing group composition and how the dynamic change, it’s necessary to provide specific tools.

Many analytic models and simulation of aggregation (mathematical and computer-based) offer interesting tools to investigate aggregation phenomena in various species (Schellinck and White 2011), including scale of different aggregate-level behaviour (individuals, castes, groups, or species).

Description of animal movements in their environment is necessary to understand species, dispersion strategy as results of individual intrinsic factors, collective responses and social relationship (including change of individual composition moving in or out the group) and also to predict their geographical needs and spatial distribution, providing consistent data for models and simulation accuracy (Schellinck and White 2011).

In order to study aggregation patterns, researchers can use various methods for data collection that could be divided into three mains categories (1) manual : observation and capture (Krause et al. 2000; Spieler 2003), photography and film (Aschwanden et al. 2008; Ballerini et al. 2008; Boulay et al. 2013; Eklund and Jensen 2011; Le Goff et al. 2009; Yoshida et al. 2010) (2) semi-manual : sonar and echo sound (Axelsen et al. 2001; Gerlotto et al. 2006; Gerlotto and Paramo 2003; Handegard 2007; Soria et al. 2003), (3) automatic : microtransponders (Jeanson 2012), RFID tagging (Planas-Sitja et al. 2015).

In the current study we propose a data processing software called NEIGHBOUR-IN which allows spatial coordinates to be attributed to individual (object) of up to three different categories (groups), such as species, sex, age, size, moult stage, etc. We illustrate the software applications with artificial patterns and experimental study of aggregation in terrestrial isopods (Crustacea, Oniscidea). The software then calculates indexes qualifying spatial distribution, composition of groups and many other parameters. All data outputs can be used for further analysis and quantification of spatial variations.

## Methods

NEIGHBOUR-IN is a software designed to analyse individuals’ coordinates belonging to up to three different groups. The software can be used only for picture analysis and not for film analysis. However film screenshots in the appropriate format could be made to follow the dynamics of the studied processes. Once the image of the objects (i.e. individuals) is loaded, the user identifies each individual by clicking on anterior and posterior extremities. Each object belongs to up to three different groups. The software includes statistical analysis and indexes to discriminate aggregates based on spatial location of individuals and their neighbours. At the end of the treatment the table of coordinates can be exported for further spatial analysis. Different displays are available in order to show the aggregates or the influence distance of each object (for example the area where the antennas can touch and interact with another individual in the case of insects).

**Figure 1.**
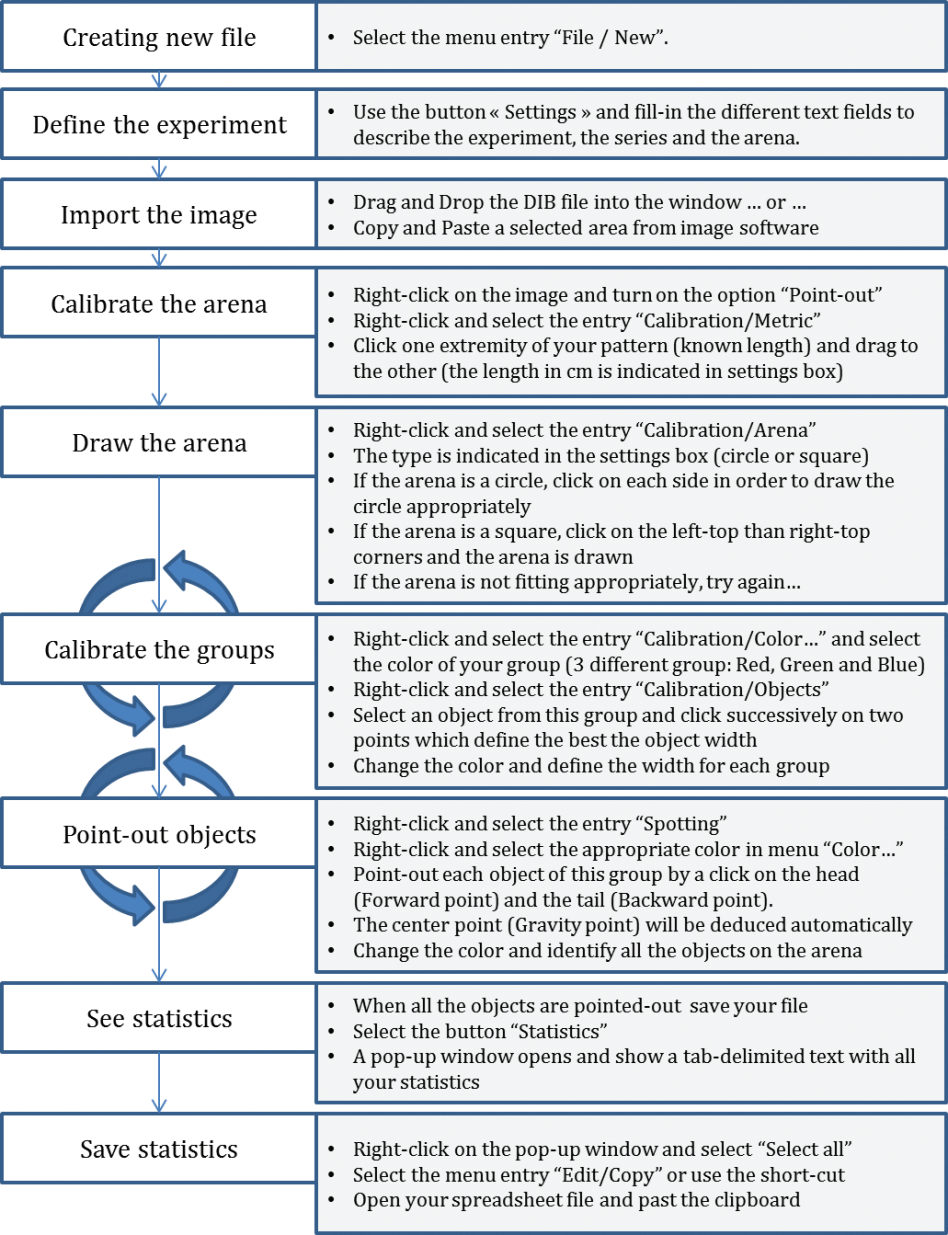
Flow chart of the creation of a new NEIGHBOUR-IN file. This figure presents the different steps in the creation of a new file, from the importation of the snapshot to the calculation of the statistics of dispersion.

Software installation and procedures (Fig. 1) are detailed in a separate additional document (NEIGHBOUR-IN Reference Manual). In the current article we will define and illustrate the different indexes of inter-individual distances and spatial distribution provided by the software and how such indexes can be helpful in the analysis of spatial distribution of objects in general and animal aggregation in particular.

Three categories of output characterising the group structure and for statistics analysis are prepared: i) inter-individual distances; ii) aggregation profile; and iii) spatial distribution.

The indexes and data included in the statistics report are:

**Header**: all the parameters of the experiment (file name; image size in pixels; experiment name; total number of objects identified; calibration information with the ratio “pixel / centimetre”, width or radius of the box and number of cells; and for each group: name, number of objects, mean body width).

**Inter-individual distance**: the mean inter-individual distances between the centre-point (G point) of all objects and both for each inter-group and intra-group combination are provided. In addition to the mean value, descriptive statistics are available in the output (sample size, standard error, minimum and maximum).

**Distance to nearest neighbours**: these descriptive statistics are identical to the ones described in the previous section (same inter-individual distances) but only between nearest neighbours (belonging to the same or different groups). The number of neighbours is defined by the user in the parameters dialog box.

**Statistics on aggregates**: When at least one of the three points of an object is included within the perception field of another object, both are considered aggregated. The perception field is calculated using the mean body width of the group multiplied by the perception ratio determined by the user for the group. The statistics include the number of aggregates automatically identified by the software; number and composition (type of group) of isolated objects; composition of each aggregate; percentage of aggregation in general and for each group (% *Aggr*.); and in the case of heterogeneous populations, Aggregation Heterogeneity Index (*AHI*). The *AHI* provides an estimate of the homogeneity of subpopulation distributions. It will be maximum (1) when the aggregates are “pure” (i.e. each one is composed by objects which belong to the same group) and minimum (0) when all individuals in the aggregates are equally mixed whatever their features. The index is calculated using the following formula:


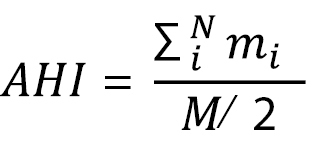


where N is the number of aggregates; m_i_ is the minimum number of objects belonging to the same group for the aggregate i; M is the total number of objects aggregated.

**Statistics on distribution**: The area is divided in different cells by the software, defining a grid. The distribution of the objects on the area is described by two indexes: the Spatial Distribution Index (*SDI*) for each group, and the Spatial Mixed Index (*SMI*) for groups’ pair comparison.

**Spatial Distribution Index** (*SDI*) is given for each group and is calculated by dividing the number of cells/sectors where at least one object of the group is present by the total number of objects in the group. *SDI* will be maximum (1) when each object is in a different cell and minimum when the objects are aggregated in few cells (0.125 for eight objects in the same cell). Therefore the *SDI* index is sensitive to the number of cells (user defined) and the size of the objects (i.e. how many objects can contain a single cell).

**Spatial Mixed Index** (*SMI*) is calculated for two groups by dividing the number of cells in which at least one object of both groups is present by the total number of cells occupied by the two groups. *SMI* will be maximum (1) when all occupied cells contain mixed groups and minimum (0) when the objects of each group are all in different cells. The *SMI* between the three groups is also computed.

**Table of distribution**: The repartition of the objects (i.e. the number of objects from each group) in the different cells/sectors of the open field is presented in a table. The number of rows/columns is defined previously by the user (parameters dialog box).

**Table of coordinates**: The list of the coordinates of each point constituting the objects are presented in a table where the objects are in rows and the type of group is in columns. Group identity number, identity number of which aggregate the object is in, the object’s width and length, and x and y coordinates in pixels for the three constitutive points (forward, gravity and backward) are included in this table.

## Illustrative examples

### Aggregation and dispersion indexes using artificial patterns

In order to validate the indexes computed by the software, we have designed artificial patterns using two groups of objects. The placement of each object has been chosen in order to reflect i) a high level of aggregation or no aggregation at all; ii) a high, medium, low or null level of inter-group affinity. The patterns are provided on the top three rows of Table 1 and illustrated by Figs 2.1–2.8. Additionally, ten replicates with random distribution of objects have been calculated. The last artificial patterns present the maxima and minima obtained in the random replicates (Table 1). The goal is to use a set of indexes able to discriminate between the different patterns.

**Figure 2.**
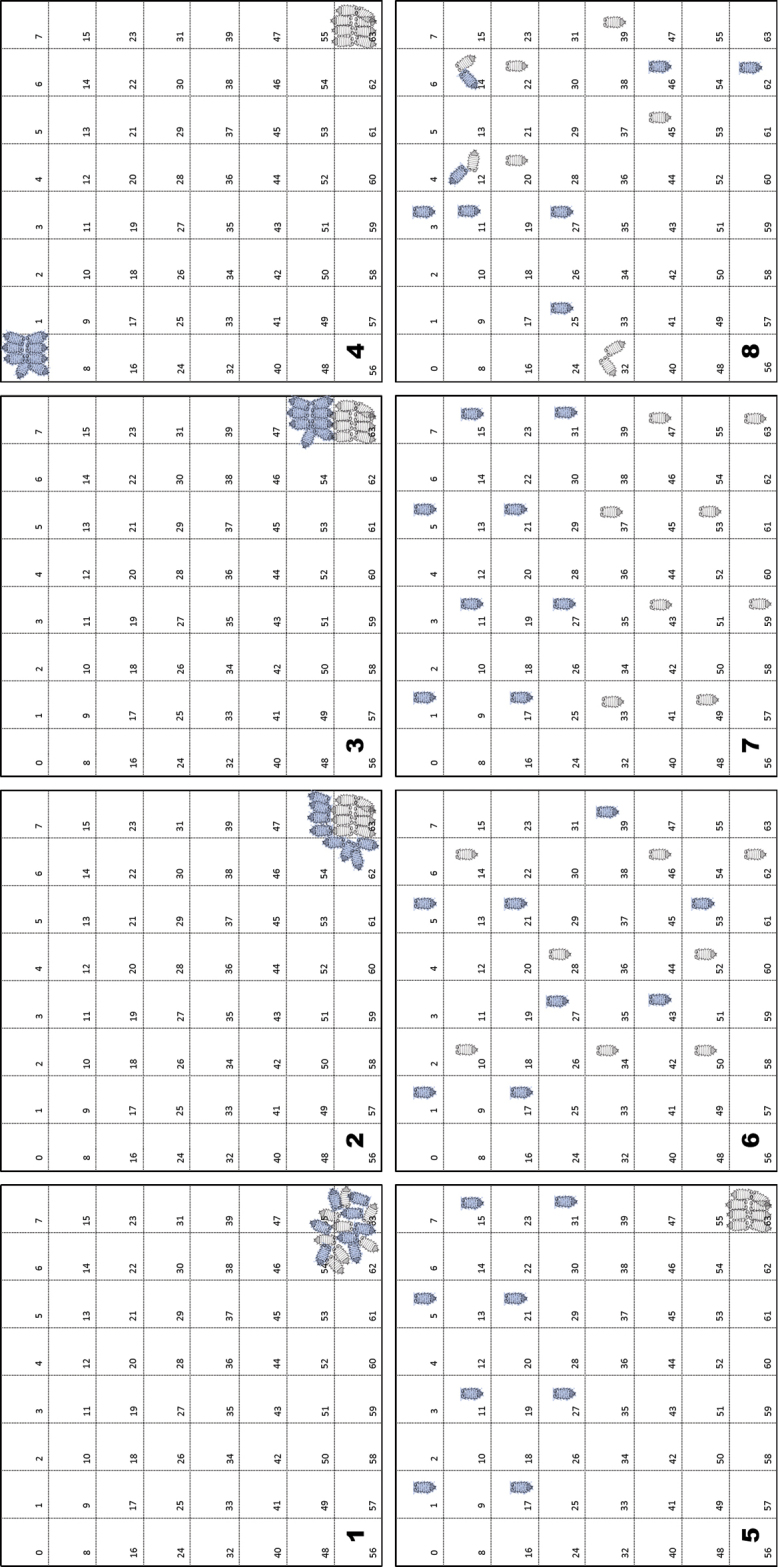
Virtual configurations used for software validation. Virtual configurations used to compile the data presented in the Table 1. Part 2.8 is one of the 10 replicates obtained with a random distribution. All other configurations have been designed in order to reach the desired level of aggregation and affinity between groups. The filled and empty shapes represented two virtual groups in the population.

**Table 1.** Evolution of indexes in different virtual configurations. Specific patterns of intra-group aggregation and inter-group affinity based on eight virtual configurations composed of two groups of 8 objects (Red and Green). The last configuration (named Random) is the average of ten replicates of a random distribution of 16 objects and presents also minima and maxima of the indexes. Each configuration is illustrated by the Figures 2.1–2.8. Categories of indexes are: i) Inter-individual distances between all objects (*All*), or objects of the red (*Red*) or green groups (*Gr.*); ii) Nearest neighbours distances considering only the three nearest neighbours; iii) Number of aggregates (*Nb Aggr.*), Aggregation heterogenity index (*AHI*); Overall aggregation level (%*Aggr. All*) and aggregation level for each group (%*Aggr. Red*) and %*Aggr. Green*); iv) Spatial distribution index (*SDI*) for each group and Spatial Mixed Index (*SMI*) between both groups.

Groups:	Configuration	Virtual 1	Virtual 2	Virtual 3	Virtual 4	Virtual 5	Virtual 6	Virtual 7	Virtual 8 (10 repl.)	
Red	Aggregation	High	High	High	High	None	None	None	Random	
Green	Aggregation	High	High	High	High	High	None	None	Random	
Red/Green	Affinity	High	Medium	Low	None	None	Medium	None	Random	
Categories of index:									Minimum	Maximum
Inter-individual Distances	All <> All	62	59	61	454	364	326	363	301	374
	Red <> All	60	66	81	819	537	318	425	304	365
	Red <> Red	63	64	40	38	293	334	292	307	451
	Red <> Gr.	60	66	81	819	537	318	425	304	365
	Gr. <> All	60	66	81	819	537	318	425	304	365
	Gr. <> Gr.	66	39	38	39	37	335	293	254	400
Nearest Neighbours Distances	All <> All	37	39	38	149	160	195	223	177	247
	Red <> All	32	46	60	799	517	175	303	164	239
	Red <> Red	49	45	31	32	219	249	211	232	344
	Red <> Gr.	32	46	60	799	517	175	303	164	239
	Gr. <> All	31	51	62	799	410	169	307	162	210
	Gr. <> Gr.	49	32	31	31	31	257	220	169	320
Aggregation	Nb Aggr.	1	1	1	2	1	0	0	0	3
	AHI	1	1	1	0	0	0	0	0	1
	%Aggr. All	100	100	100	100	50	0	0	0	43.75
	%Aggr. Red	100	100	100	100	0	0	0	0	37.50
	%Aggr. Gr.	100	100	100	100	100	0	0	0	50.00
Spatial distrib.	SDI Red	0.5	0.25	0.13	0.13	1	1	1	0	1
	SDI Gr.	0.5	0.13	0.13	0.13	0.13	1	1	0	1
	SMI Red-Gr.	1	0	0	0	0	0	0	0	0.88

Aggregation patterns can be discriminated by comparing the different indexes (see Table 1). For example, we will compare data for artificial patterns 1–4 (high aggregation) with 5–7 (high dispersion) for the red group, and between patterns 1–5 (high aggregation) and 6–7 (high dispersion) for the green group.

The three categorical values of aggregation (% *Aggr.*) reflect the overall aggregation pattern (100%, 50% or 0%) and for each group (100% or 0%) for extreme cases. The percentages of aggregation do not discriminate between two groups with a high level of aggregation, but their level of affinity differs.

The Aggregation Heterogeneity Index (*AHI*) can be used to discriminate between configurations where both groups are highly aggregative according to the presence or absence of affinity between them.

The Number of Aggregates index (*Nb Aggr.*) will show the exact number of aggregate but without providing affinity information (except in case of null affinity). When the two groups are not aggregating in the same way, the index is similar (for example between configurations 3 and 5). In our example group affinity will be characterised more using the spatial distribution index.

The Spatial Distribution Index (*SDI*) reflects the different aggregation patterns for both groups. However, in the case of high affinity between groups, the index increases significantly.

The other index of this category, the Spatial Mixed Index (*SMI*), reflects the high affinity in configuration 1. However, such an index is similar in the other configurations. These two indexes of spatial distribution are complementary to other indexes.

In the case of non-aggregative groups, showing or not a relative affinity (for example configuration 6 or 7 respectively), neither the percentage of aggregation nor the number of aggregates differs. In this case (see Fig. 2.6 and 2.7) the comparison of inter-individual distances will be the most informative to characterise individual affinity.

Inter-individual distance is a good indicator of aggregation level. Intra-group inter-individual distances are minimal when aggregation level is high. However, Table 1 shows that when affinity is high between groups, such intra-group inter-individual distances increase, as immediate neighbours can belong to both groups due to affinity (configurations 1–2). Use of distances to nearest neighbours limits this inconvenience. The inter-group distances are good indicators of the relationship between groups. Finally, the different levels of affinity between groups differentiate these two configurations.

### Case study: Aggregation in Woodlice

The indexes have been tested using snapshots of individual distribution in terrestrial crustacean species (Oniscidea). Woodlice are good candidates for aggregation studies since such behaviour is widespread in this group and is explained as an adaptive response supporting their conquest of terrestrial life (Broly et al. 2013; Broly et al. 2012; Caubet et al. 1998; Caubet et al. 2008) and is under social component (Beauché and Richard 2013; Devigne et al. 2011). Such crustaceans present several specific constraints (weakness, group density. etc.) which lead to difficulties in real time tracking in comparison to insects.

**Figure 3.**
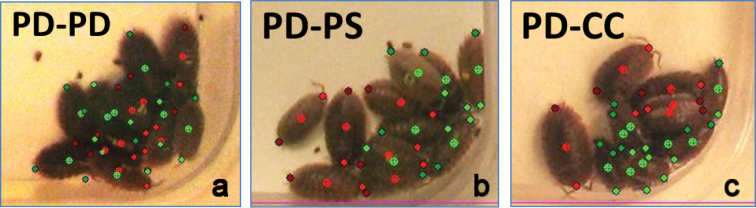
Aggregation heterogeneity in woodlice. Aggregation patterns of two groups of woodlice illustrating the Aggregation Heterogenity Index (*AHI*) and the Spatial Mixed Index (*SMI*). PD: *Porcellio dilatatus*, PS: *Porcellio scaber*, CC: *Cylisticus convexus*. Values of indexes: PD-PD: *AHI*=0.93 & *SMI*=0.80; PD-PS: *AHI*=0.67 & *SMI*=0.60; PD-CC: *AHI*=0.63 & *SMI*=0.33.

**Figure 4.**
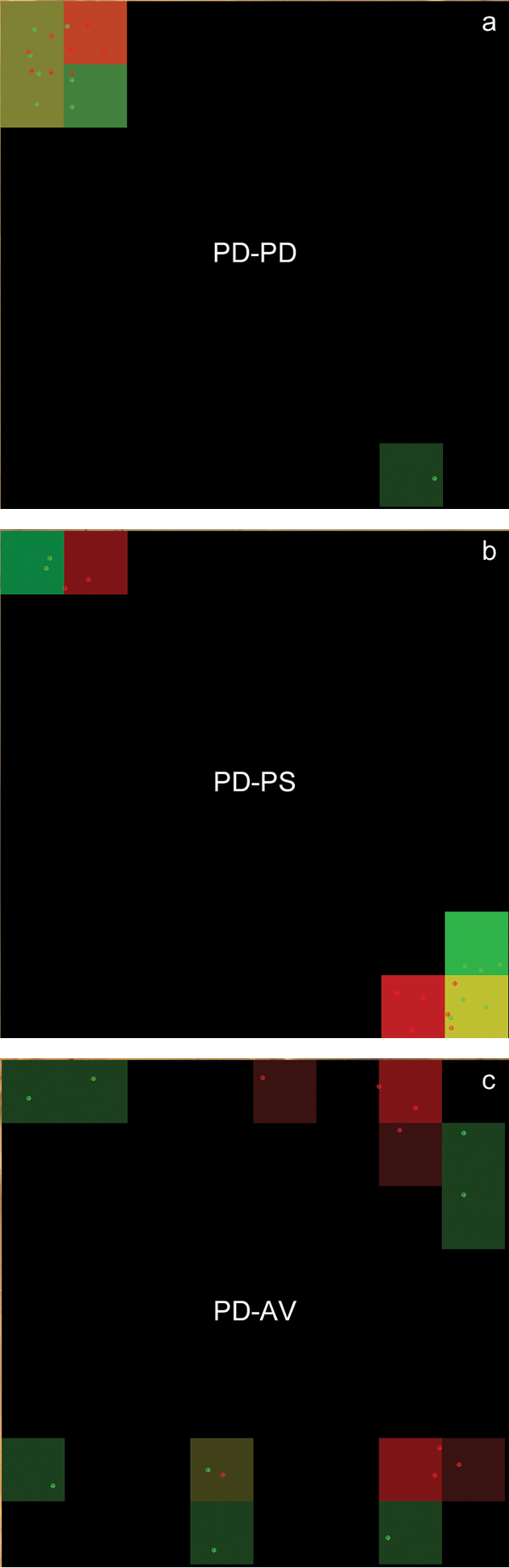
Spatial distribution in woodlice. Graphic outputs of spatial distribution patterns obtained in three configurations with monospecific or bispecific populations including two groups of eight individuals: **a** PD-PD: The two groups are *Porcellio dilatatus* (red and green) **b** PD-PS: *Porcellio dilatatus* (red) and *Porcellio scaber* (green) **c** PD-AV: *Porcellio dilatatus* (red) and *Armadillidium vulgare* (green). The outputs show 64 cells. Each cell is represented with a colour corresponding to the individual(s) in that cell. The colour is mixed using green and red proportional to the number of green and red individuals. If the cell is empty, the colour is black. The intensity of the colour reflects the number of individuals. The position of the individual is determined by its point G (centre-point).

First, we compared three gregarious species (groups): *Porcellio dilatatus* (PD), *Porcellio scaber* (PS) and *Cylisticus convexus* (CC). Three different combinations of two groups of eight individuals (“objects”) are placed in a squared arena (width 12.3 cm) divided into 64 cells. After one hour, a snapshot is taken (Fig. 3) and the distribution of the individuals is analysed with NEIGHBOUR-IN. We used indexes in order to characterise our aggregates according to group characteristics. In a second step, we added the species *Armadillidium vulgare* (AV) to PD and PS. This species presents a more scattered aggregation pattern (Hassall et al. 2010). A snapshot is taken after one hour and analysed. The indexes are presented in Table 2 and the outputs concerning spatial distribution in Fig. 4 (“*Surfaces*” output display).

**Table 2.** Evolution of indexes in real configurations. Specific patterns of intra-group aggregation and inter-group affinity based on three real configurations composed of two groups of 8 woodlice (Red and Green). PD: *Porcellio dilatatus*; PS: *Porcellio scaber*; AV: *Armadillidium vulgare*. Each configuration is illustrated by figs 4.a-4.c. See Table 1 for the description of the categories of indexes.

Groups	Configuration	PD-PD	PD-PS	PD-AV
Species	Red	PD	PD	PD
	Green	PD	PS	AV
Categories of index:				
Inter-individual Distances	All <> All	188	174	541
	Red <> All	188	183	550
	Red <> Red	71	279	449
	Red <> Gr.	188	183	550
	Gr. <> All	188	183	550
	Gr. <> Gr.	305	50	614
Nearest Neighbours Distances	All <> All	113	107	302
	Red <> All	49	160	288
	Red <> Red	53	217	305
	Red <> Gr.	49	160	288
	Gr. <> All	155	54	321
	Gr. <> Gr.	130	37	492
Aggregation	Nb Aggr.	1	2	4
	AHI	0.93	0.67	0.20
	%Aggr. All	93.75	93.75	62.50
	%Aggr. Red	100.00	87.50	87.50
	%Aggr. Gr.	87.50	100.00	37.50
Spatial distrib.	SDI Red	0.500	0.500	0.75
	SDI Gr.	0.625	0.375	1
	SMI Red-Gr.	0.800	0.400	0.077

In our combinations, a first analysis focused on the main aggregate obtained in each combination using *AHI* and *SMI* indexes as descriptors of the quality of the aggregates (Fig. 3). In these snapshots the aggregate is composed of animals from both groups. However, the pattern of aggregation is different and the inter-individual distances cannot be used, since all individuals are close to one other. However, the use of *AHI* and *SMI* indexes can be informative since they differentiate the three combinations. When the two groups belong to the same species *Porcellio dilatatus* (PD-PD, Fig. 3.a), affinity score is at a maximum between individuals and the *AHI* and *SMI* are at their highest score (respectively 0.93 and 0.80). When both groups belong to the species *Porcellio dilatatus* and *Cylisticus convexus* (PD-CC, Fig. 3.c), the *AHI* and *SMI* are at their lowest (respectively 0.63 and 0.33). The intermediary configuration with the species *Porcellio dilatatus* and *Porcellio scaber* (PD-PS, Fig. 3.b) shows intermediary indexes (respectively 0.67 and 0.60). In conclusion, even in the case of very aggregative species, the quality of the aggregation pattern, matched with the affinity between individuals, can be characterised using a combination of complementary indexes.

The distribution of the two groups and the aggregation level appear to be very different according to the species pairing (Fig. 4). The indexes computed by NEIGHBOUR-IN reflect qualitative and quantitative differences in aggregation pattern variability well (see indexes on Table 2).

In the homospecific combination of the species *Porcellio dilatatus* (PD-PD, Fig. 4.a) a single individual in the green group is isolated while all other individuals are crowded in a single mixed aggregate (five cells among 64 contain individuals). Only the cell containing the isolated individual is pure (bottom right corner on Fig. 4.a), while the four other cells contain animals that belong to both groups (*SMI* = 0.8; most of the cells contain individuals from both groups) and the distribution is totally mixed (*AHI* = 0.93; higher level of heterogeneity in the aggregate). The inter-individual distances show differences between red and green groups due to the isolated green individual (71 and 305 for reds and greens respectively). The nearest neighbour distances adjust the values especially for the green group (53 and 130 for reds and greens respectively). The intra- and inter-group distances are similar (53 and 49 pixels respectively). Aggregation indexes reflect the high level of aggregation of both groups (between 100% and 87.5% for reds and greens respectively). Concerning the spatial distribution index (*SDI*), the fact that one individual is isolated in the green group induces a small difference in the index (0.5 and 0.625 for red and green groups respectively).

In the heterospecific combination, with the species *Porcellio dilatatus* – *Porcellio scaber* (PD-PS, Fig. 4.b), we obtain two distinct aggregates located in the two opposed corners of the arena. Even if both species present a high level of aggregation (87.5% and 100% for PD and PS), the indexes are able to differentiate the quality of aggregation in comparison to the homospecific configuration (PD-PD). Both *AHI* and *SDI* values decrease (0.67 and 0.4 respectively). We observed that individuals in the same aggregate are sharing a single cell (Fig. 4.b). The other individuals are juxtaposed but not mixed, and stay close to conspecifics. Among the five occupied cells, four of them are occupied by individuals of the same group. Inter-individual distances increase because the aggregates are separated. The nearest neighbour distances adjust the values, and we observe an inter-group distance higher than in the homospecific configuration (169 rather than 49).

The third combination, using the two species *Porcellio dilatatus* and *Armadillidium vulgare* (PD-AV, Fig. 4.c) presents another pattern of distribution: The green group (*Armadillidium vulgare*) appears less aggregative than the red group (*Porcellio dilatatus*) (37.5% and 87.5% respectively). In this configuration, four aggregates are identified by the software. The *AHI* index, reflecting the mixture of the aggregates, is very low (0.2) compared to homospecific and genera-related configurations (0.93 and 0.67 respectively). Moreover, the spatial distribution is completely different for both species: *Armadillidium vulgare* shows the maximum value of *SDI* index (1.0) meaning that each individual is in a different cell, and *Porcellio dilatatus* shows a more dispersed distribution (0.75) than in the other two combinations (PD *vs.* PD and PD *vs.* PS) (0.5).

## Discussion

Our image processing software, NEIGHBOUR-IN, provides indices with efficient discriminatory power to characterize and analyse group structure using individuals’ coordinates. In the features of the software we integrate elementary statistical analysis and complementary index calculation that are important tools to describe aggregations, as well as a new index for more precise analysis. We provided examples based on random data and a case study using gregarious arthropods to highlight the accuracy of the output information. Moreover, the raw data, individual coordinates and location can be directly manipulated by the researcher for specific analysis such as simulation, modelling, and classic spatial statistics.

One of the assets of NEIGHBOUR-IN is the distinction between up to three groups and the open group size, which allow for a variety of applications. The differentiation between groups of individuals can be applied to compare intra-specific and inter-individual affinity according to size, age, sex, moult stage, health, and genetic relatedness at the individual level. Behavioural adaptive responses in inter-specific interactions is also an important field of investigation, and NEIGHBOUR-IN could be a new tool to study prey-predator, host-parasite, and commensalism impacts on group formation and composition. How the dispersion of animals in heterogeneous habitat and physical environment changes the aggregation pattern can also be investigated with this method. Moreover, the management of the image doesn’t require a specific template or scale, and can be used with all type of images including aerial images of vertebrates, to macro photography of small invertebrates, and even picture under microscope with micro-organisms.

In comparison with the tools available in aggregation analysis, NEIGHBOUR-IN appears to be an accessible, light, and open solution. Our software is designed for analysis at a point-time however continuous monitoring of behavioural is not possible. The level of integration and data analysis complexity is smaller in comparison to GIS systems, which require user training and specific data templates and libraries. Other powerful softwares that track and analyse animal movement, such as Noldus ETHOVISION, analyse in real time and are not designed for snapshot analysis. Noldus ETHOVISION analyses are automated and qualitative information on aggregates could be missed. Many species present complex aggregate structures and often in three dimensions so that a fully automatic tracking is necessarily imprecise. For example, when two individuals are superimposed, softwares like Noldus ETHOVISION lose track of the two individuals (one source of imprecision) and then randomly assign the initial characteristics to individuals when they separate, so that both intermediate and final results can lead to incoherency. Our semi-manual software allows manual localisation of individuals, increasing the precision of the spatial encoding, while keeping an automatic acquisition of results and analyses. Moreover, the user is able to identify orientation of individual (anterior and posterior extremities) with its perception area and consequently the possibility of interactions or not.

Finally, this software promises to evolve with new features, and could be used to generate and export distribution and coordinates database for other purposes. Potential fields of applications could be evolution of invasive species (animals and plants) distribution using aerial image, competition of fungus or microbial colonies, identification of harems in marine mammals grouping and so on.

The main limitations of NEIGHBOUR-IN software are in the potential excessive overlap of the individuals in an aggregate, and the total number of group and individuals taken into account. However, in both case, the user himself/herself is confronted to difficulties and the task, even if it is complicated, will be easier using NEIGHBOUR-IN. In comparison to a direct analysis of the image, the advantage to generating NEIGHBOUR-IN data files is that the image is saved with the coordinates but the statistics are managed separately, which allows the researcher to re-use the same files with further statistical analysis, as well as the integration of new graphic outputs and indexes.
